# Early childhood aggressive behaviour: Negative interactions with paternal antisocial behaviour and maternal postpartum depressive symptoms across two international cohorts

**DOI:** 10.1016/j.eurpsy.2018.07.007

**Published:** 2018-10

**Authors:** Mijke P. Lambregtse-van den Berg, Henning Tiemeier, Frank C. Verhulst, Vincent Jaddoe, Elizabeth Tindall, Haido Vlachos, Katie Aumayer, Jane Iles, Paul G. Ramchandani

**Affiliations:** a Department of Child and Adolescent Psychiatry/Psychology, Erasmus MC-University Medical Centre, Rotterdam, The Netherlands; b Department of Psychiatry, Erasmus MC-University Medical Centre, Rotterdam, The Netherlands; c Department of Social and Behavioral Science, Harvard TH Chan School of Public Health, Boston, United States; d Department of Epidemiology, Erasmus MC-University Medical Centre, Rotterdam, The Netherlands; e The Generation R Study Group, Erasmus MC-University Medical Centre, Rotterdam, The Netherlands; f Department of Pediatrics, Erasmus MC-University Medical Centre, Rotterdam, The Netherlands; g Department of Psychiatry, University of Oxford, United Kingdom; h Milton Keynes Child and Adolescent Mental Health Service, CNWL NHS Trust, Milton Keynes, United Kingdom; i Academic Unit of Child and Adolescent Psychiatry, Imperial College, London, United Kingdom; j School of Psychology, University of Surrey, United Kingdom; k PEDAL Centre, Faculty of Education, University of Cambridge, United Kingdom

**Keywords:** Paternal antisocial behaviour, Maternal depression, Child aggressive behaviour, Cohort studies

## Abstract

**Background:**

Early childhood aggressive behaviour is a predictor of future violence. Therefore, identifying risk factors for children’s aggressive behaviour is important in understanding underlying mechanisms. Maternal postpartum depression is a known risk factor. However, little research has focused on the influence of paternal behaviour on early childhood aggression and its interaction with maternal postpartum depression.

**Methods:**

This study was performed in two cohorts: the Fathers Project, in the United Kingdom (n = 143) and the Generation R Study, in The Netherlands (n = 549). In both cohorts, we related paternal antisocial personality (ASP) traits and maternal postpartum depressive (PPD) symptoms to childhood aggressive behaviour at age two (Fathers Project) and age three (Generation R Study). We additionally tested whether the presence of paternal ASP traits increased the association between maternal PPD–symptoms and early childhood aggression.

**Results:**

The association between paternal ASP traits and early childhood aggressive behaviour, corrected for maternal PPD-symptoms, was similar in magnitude between the cohorts (Fathers Project: standardized β = 0.12, p = 0.146; Generation R: β = 0.14, p = 0.001), although the association was not statistically significant in the Fathers Project. Strikingly, and in contrast to our expectations, there was evidence of a negative interaction between paternal ASP traits and maternal PPD-symptoms on childhood aggressive behaviour (Fathers Project: β = −0.20, p = 0.020; Generation R: β = −0.09, p = 0.043) in both studies. This meant that with higher levels of paternal ASP traits the association between maternal PPD-symptoms and childhood aggressive behaviour was less and vice versa.

**Conclusions:**

Our findings stress the importance of including both maternal and paternal psychopathology in future studies and interventions focusing on early childhood aggressive behaviour.

## 1 Introduction

Aggression and violence are a worldwide major health concern [1]. Childhood aggressive behaviour is one of the strongest predictors of adverse outcomes in adolescence, including school dropout, substance abuse, crime and unemployment [2,3]. Children who show early onset and life-course-persistent aggressive behaviour are at highest risk for serious violent acts in adulthood, compared to late-onset (early adulthood) aggressive behaviour [4,5]. Therefore, identifying risk factors for early childhood aggressive behaviour is important for understanding underlying mechanisms and to inform the development of preventive interventions. Neurobiological and environmental risk factors that have been associated with early child aggressive behaviour include: exposure to maternal prenatal substance use (tobacco, alcohol, drugs), birth complications, malnutrition, lead exposure, childhood head injury, maternal psychopathology and adverse parent-child interactions (e.g. abuse, neglect) [6,7].

With respect to the influence of parental psychopathology on early child aggressive behaviour, more attention has been paid to maternal psychopathology, and in particular maternal depression, compared to paternal psychopathology [6]. This focus might be explained by the high prevalence of maternal postpartum depression, with prevalence rates between 5–20% in the first 3 months after delivery [8], and the fact that in many societies women still are the primary care givers for children suggesting that they have a more crucial role in early child development than men. Indeed, previous research showed that maternal postpartum depression strongly predicts early childhood aggressive behaviour [9]. However, the potential influence of paternal antisocial behaviour on early childhood aggressive behaviour is of particular interest, because antisocial behaviour is more prevalent in men compared to women, has a strong heritability [10] and is likely to be transmitted to the child independently from maternal depression and through other mechanisms. For example, previous research showed that 40–70% of the variance in children’s aggressive behaviour could be explained by genetic factors [11,12]. In addition to genetic factors other related environmental influences may be important, including harsh parenting, increased couple conflict, poverty and substance use [13].

Therefore, it is important to consider the role of paternal antisocial behaviour in the development of childhood aggressive behaviours, alongside the established association with maternal depression. As well as individual effects, these different risk factors may interact. For example, the presence of paternal antisocial behaviour might modify the effect of maternal postpartum depression on early childhood aggressive behaviour. A meta-analysis showed preliminary evidence that paternal psychopathology does increase the risk of externalizing behaviour in school-aged children of mothers with depression [14].

However, little is known about the interaction between maternal depression and paternal antisocial behaviour on aggressive behaviour in preschool children. We are aware of one relatively small study (n = 101), which showed that paternal psychopathology (67% mood and/or anxiety disorder and 23% substance use and/or antisocial behaviour disorders) moderated the association between a history of maternal depression on toddlers' externalizing behaviour problems [15]. In this study maternal depression was significantly associated with toddlers’ externalizing behaviour problems only when paternal psychopathology was also present. This finding requires replication.

The aim of our study was to investigate whether paternal antisocial personality (ASP) traits are associated with early childhood aggressive behaviour and to which extent this association is influenced by the occurrence of maternal postpartum depressive (PPD) symptoms. We hypothesised that, after adjustment for maternal PPD-symptoms, paternal ASP traits would be associated with early childhood aggressive behaviour. Additionally, we tested the hypothesis that there would be a positive interaction between paternal ASP traits and maternal PPD-symptoms on early childhood aggressive behaviour, assuming that presence of paternal ASP traits would increase the association between maternal PPD-symptoms and early childhood aggressive behaviour. We used data from two different European cohorts to investigate cross-cohort consistency of any observed associations.

## 2 Materials and methods

### 2.1 Design and participants

This study uses data from the Fathers Project and the Generation R Study. The Fathers Project is a longitudinal study of fathers and their families. Participants were recruited from the postnatal maternity wards of hospitals in Oxford and Milton Keynes, UK. They were assessed at home at 3 months, 1 year and 2 years postpartum. The initial recruitment process has been described in more detail elsewhere [16]. Children were required to have been born at no less than 37 weeks, to have a birth weight of at least 2,500 g and to have no severe illness or abnormalities. In total 192 families were included. In 143 families complete data was available on all variables and at least a father or a mother report of early childhood aggressive behaviour at age two.

The Generation R Study is a longitudinal population-based study conducted in Rotterdam, The Netherlands, and follows children and their family from foetal life onwards. It has been described in detail elsewhere [17,18]. To make the study population more comparable with the Fathers Project, we excluded families with children born less than 37 weeks and a birth weight of less than 2,500 g (n = 42). In 542 families complete data were available on all variables and at least a father or a mother report of early childhood aggressive behaviour at age three.

Both studies were approved by the local Medical Ethics Committees, and informed consent was obtained from all participants.

### 2.2 Paternal antisocial behaviour

Fathers Project: Fathers’ antisocial personality traits were measured at inclusion (3 months postnatally) with the Antisocial Personality Problems scale from the Adult Self-Report DSM-oriented scales [19]. The scale consists of 20 items, with a response scale from 0= not true to 2= very true or often true (range 0–40).

Generation R: Fathers’ antisocial personality traits were measured with the National Institute of Mental Health Diagnostic Interview Schedule (DIS), which included antisocial personality [20]. Trained interviewers conducted the interview in a home visit around 30 weeks of pregnancy. We used the number of fulfilled A criteria of the DSM-IV antisocial personality disorder as a continuous measure (range 0–7).

### 2.3 Maternal postpartum depressive symptoms

In both the Fathers Project and the Generation R Study maternal PPD-symptoms were assessed with the Edinburgh Postnatal Depression Scale (EPDS) a 10 item widely used self-report questionnaire (range 0–30), that has also been validated in Dutch [21,22]. In the Fathers Project the EPDS was measured at 3 months after childbirth and in the Generation R Study the EPDS was assessed at 2 months after childbirth.

### 2.4 Early childhood aggressive behaviour

In both studies the Child Behavior Checklist/1½-5 (CBCL/1½-5) was used for parental reports of child behaviour problems [19]. This questionnaire contains 99 items, which are scored on a three-point scale from 0= not true to 2= very true or often true, based on the 2 preceding months. The CBCL/1½-5 was filled out by both parents at children’s age of 2 years in the Fathers Project and at age of 3 years in the Generation R Study. For this study we used the Aggressive Behavior syndrome scale, which comprised items such as: ‘destroys things’, ‘cruel to animals’, and ‘gets in many fights’. For our secondary analyses to examine the specificity of our findings we used the broadband Internalizing Scale consisting of the sum score of the items (N = 24) of the four syndrome scales: Emotionally Reactive, Anxious/Depressed, Somatic Complaints, and Withdrawn.

To obtain a score on behaviour problems based on the report of both parents, the scores were first standardized (Z-scores) and then averaged. If only the score of one parent was available this score was used (5% in the Fathers Study and 8% in the Generation R Study).

### 2.5 Covariables

Fathers Project: Information on paternal age, educational level and child gender were obtained during inclusion at 3 months after childbirth. Educational level was categorized in three levels: low (no qualification/GCSE/A level), middle (Diploma or equivalent) and high (Degree/Postgraduate degree).

Generation R Study: Information on paternal age and educational level were obtained at inclusion around 12 weeks pregnancy. Educational level was categorized in three levels: low (primary school and lower vocational education), middle (intermediate vocational education) and high (higher vocational education and university). Child gender was obtained from midwife and hospital registries.

### 2.6 Statistical analyses

In the non-response analyses we analysed differences between baseline characteristics of responders and non-responders on the assessment of early childhood aggressive behaviour problems with Chi-squared test for educational level, the independent *t*-test for paternal age and the Mann-Whitney U test for non-normally distributed continuous variables.

Linear regression was used to assess the influence of ASP traits and maternal PPD-symptoms on early childhood aggressive behaviour. Paternal ASP traits and maternal PPD-symptoms were analysed as continuous measures. These measures were centred around the mean to avoid problems with multicollinearity and improve interpretation.

We adjusted for paternal age and educational level, since our main interest was on the influence of paternal psychopathology corrected for paternal characteristics on early childhood aggressive behaviour. In the first step we separately investigated the main effects of ASP traits and maternal PPD-symptoms adjusted for main paternal confounding variables. In the second step we adjusted the analyses for psychopathology of the other parent to examine independent effects of ASP traits and maternal PPD-symptoms. In the third step we included the interaction effect of paternal ASP traits and maternal PPD-symptoms. To test the specificity of the findings for the influence of paternal ASP traits and maternal PPD-symptoms on early childhood aggressive behaviour, we repeated the same analyses with early childhood internalizing behaviour as a continuous measure.

In all statistical analyses the level of significance was set at α = 0.05. Statistical analyses were performed with the Statistical Package of Social Sciences version 20.0 for windows.

### 2.7 Non-response analyses

Fathers Project: Families with loss to follow up or missing data (n = 49; 26%) significantly differed from families who were included in the analyses on maternal PPD-symptoms [median (90% range) 7 in those with missing data (1.4–12.6) versus 5 (1.0–10.6); Z= −2.46; p = 0.01] and child gender (63.3% female versus 42.0% male ; X^2^ = 6.65; p = 0.01). Paternal age, paternal educational level, paternal ASP traits and both father and mother report on early childhood aggressive behaviour did not significantly differ between groups.

Generation R: Families lost to follow up or with missing data (n = 282; 34%) did not significantly differ from families who were included in the analyses on paternal educational level, age, paternal ASP traits, maternal PPD-symptoms and both father and mother report on early childhood aggressive behaviour.

## 3 Results

Table 1 presents the characteristics of both cohorts. In both cohorts the participating fathers were mostly highly educated. The level of maternal PPD-symptoms and early childhood aggressive behaviour was slightly higher in the Fathers Project compared to the Generation R Study, while the level of paternal education and paternal ASP traits were slightly higher in the Generation R study. However, comparison of the level of paternal ASP traits is difficult, because different assessment scales were used. In both samples the occurrence of ASP symptoms was low (mean score of 3.3 and median score of 2 out of possible range of 0–50 within the Fathers Project and mean score 1.1 and median score of 0 out of range 0–7 within the Generation R Study).

**Table 1:** Sample characteristics.

	Fathers project (N = 143)		Generation R (N = 542)		test statistics	p
Child gender (N; %)						
*Male*	60	42	267	49.3	Chi2(1)= 2.419	0.012
*female*	83	58	275	50.7		

Paternal age (mean; SD)	34.9	5.78	33.8	4.74	T(683)= 2.352	0.468

Paternal educational level (N; %)						
*low*	29	20.3	65	12	Chi2(2)= 6.739	0.034
*middle*	26	18.2	118	21.8		
*high*	88	61.6	359	66.2		

Paternal antisocial personality traits (median; 90% range)^a^	2	(0–6.6)	0	(0–3)	STS= −9,399	<0.001

Maternal depressive symptoms (median; 90% range)	5	(1–10.6)	3	(0–9)	STS= −6,017	<0.001

Child aggressive behaviour						
maternal report (median; 90% range)	9	(3–14.9)	5.3	(1–13)	STS= −5,971	<0.001
paternal report (median; 90% range)	9	(3–16)	7	(1–15)	STS= −4,206	<0.001

SD = standard deviation.

STS=Standardized Test Statistic.

a Different numbers are explained by different measures that were used to assess paternal antisocial personality traits.

### 3.1 Paternal antisocial personality traits and maternal postpartum depressive symptoms

Table 2 presents associations between paternal ASP traits and early childhood aggressive behaviour. After adjustment for relevant variables and psychopathology in the other parent, in the Fathers Project there was no significant association between paternal ASP traits and early childhood aggressive behaviour (standardised β = 0.12; p = 0.146), although the effect of paternal ASP traits in the adjusted analyses was similar with the association we found in Generation R (β = 0.14; p = 0.001).

**Table 2:** The association between paternal ASP and maternal PPD on early childhood aggressive behaviour (combined father-mother report).

			*Model 1*^a^					*Model 2*^b^					Model 3^c^		
	Standardized beta	B	95% CI	t	p	Standardized beta	B	95% CI	t	p	Standardized beta	B	95% CI	t	p
**Fathers project (N = 143)**															
Paternal antisocial behaviour	0.14	0.04	(−0.01; 0.10)	1.69	0.093	0.12	0.04	(−0.01; 0.09)	1.46	0.146	0.16	0.05	(−0.03; 0.10)	1.88	0.063
Maternal depressive symptoms	**0.18**	**0.04**	**(0.002; 0.07)**	2.08	**0.039**	**0.19**	**0.04**	**(0.004; 0.08)**	**2.21**	**0.029**	**0.23**	**0.05**	**(0.01; 0.09)**	**2.65**	**0.009**
Paternal ASP * Maternal PPD											**−0.20**	**−0.01**	**(**−**0.02;** −**0.002)**	**−2.35**	**0.020**

**Generation R (N = 549)**															
Paternal antisocial behaviour	**0.14**	**0.09**	**(0.03; 0.14)**	**3.25**	**0.001**	**0.14**	**0.09**	**(0.03; 0.14)**	**3.27**	**0.001**	**0.15**	**0.09**	**(0.04; 0.14)**	**3.46**	**0.001**
Maternal depressive symptoms	0.06	0.01	(−0.007; 0.04)	1.29	0.197	0.05	0.01	(−0.008; 0.04)	1.22	0.220	0.06	0.02	(−0.005; 0.04)	1.50	0.134
Paternal ASP * Maternal PPD											**−0.09**	**−0.01**	**(**−**0.02; <0.01)**	**−2.03**	**0.043**

Bold values represent statistical significance *p* < 0.05.

a Adjusted for: paternal age, educational level father, infant gender (psychopathology of the other parent not included).

b As model I with additional adjustedment for psychopathology of the other parent.

c As model II with inclusion of interaction term.

In the Fathers Project we found an association between maternal PPD-symptoms and aggressive early childhood behaviour (β = 0.19; p = 0.029). In the Generation R Study this association was not present (β = 0.05; p = 0.220).

### 3.2 Interaction between paternal ASP traits and maternal PPD-symptoms

In both cohorts there was a negative interaction between paternal ASP traits and maternal PPD-symptoms on early childhood aggressive behaviour (Fathers Project: standardized β= −0.20; p = 0.020 and Generation Study: β= −0.09; p = 0.043). Although the effect of the interaction was more pronounced in the Fathers Project than in the Generation R study, our results consistently showed that with higher paternal ASP traits the association between maternal PPD-symptoms and early childhood aggressive behaviour was less. Alternatively, with higher levels of maternal PPD-symptoms the association between paternal ASP traits and early childhood aggressive behaviour was less.

**Fig. 1:**
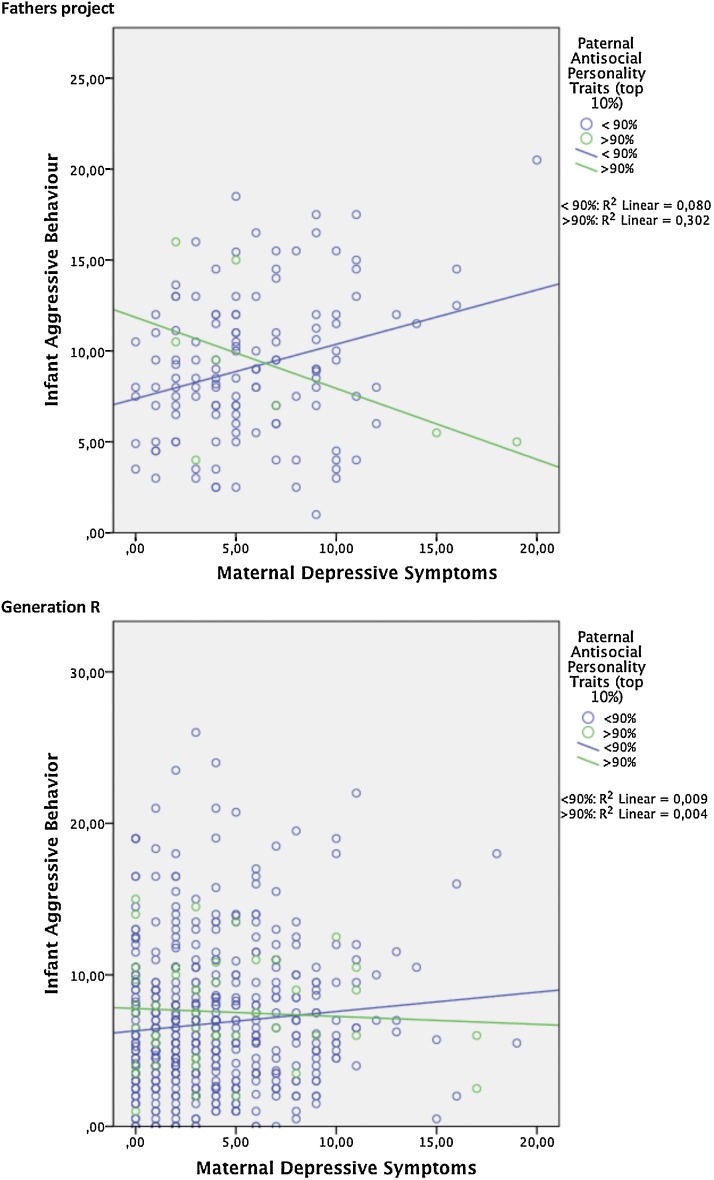
Association between maternal depressive symptoms and infant aggressive behaviour in fathers scoring below and above the 90% cut off score for antisocial personality traits*. *For interpretation raw scores are plotted. In the regression analyses Z-scores were used.

The interaction between paternal ASP traits and maternal PPD-symptoms is shown in Fig. 1. In this figure paternal ASP traits were dichotomized in fathers scoring above and below the 90% threshold of ASP traits. When fathers had lower scores on ASP traits there was a positive association between maternal PPD-symptoms and early childhood aggressive behaviour. However, when fathers had high scores on ASP traits there was a negative association between maternal PPD-symptoms and early childhood aggressive behaviour. These associations were present in both cohorts, though less pronounced in the Generation R Study.

### 3.3 Specificity of the findings

In Table 3 the associations between paternal ASP traits and maternal PPD-symptoms on early childhood internalizing behaviour are presented. In both cohorts there was no significant association between paternal ASP traits and early childhood internalizing behaviour (Fathers Project: standardized β = 0.03; p = 0.718 and Generation Study: β = 0.07; p = 0.153). However, we observed an association between maternal PPD-symptoms and early childhood internalizing behaviour (Fathers Project: β = 0.18; p = 0.038 and Generation Study: β = 0.12; p = 0.008). In both cohorts there was no evidence for an interaction between paternal ASP traits and PPD-symptoms in the relation with internalizing behaviour (Fathers Project: β = 0.01; p = 0.933 and Generation Study: β= -0.03; p = 0.588).

**Table 3:** The association between paternal ASP and maternal PPD on early childhood internalizing behaviour (combined father-mother report).

			*Model1*^a^					*Model2*^b^					*Model 3*^c^		
	Standardized beta	B	95% CI	t	p	Standardized beta	B	95% CI	t	p	Standardized beta	B	95% CI	t	p
**Fathers project (N = 132)**															
Paternal antisocial behaviour	0.03	0.01	(−0.04; 0.06)	0.36	0.718	0.02	0.01	(−0.05; 0.06)	0.19	0.848	0.02	<0.01	(−0.05; 0.06)	0.18	0.856
Maternal depressive symptoms	**0.18**	**0.04**	**(0.02; 0.08)**	**2.10**	**0.038**	**0.18**	**0.04**	**(0.002; 0.08)**	**2.07**	**0.041**	0.18	0.04	(0.002; 0.08)	1.89	0.061
Paternal ASP * Maternal PPD											0.01	<0.01	(−0.01; 0.01)	0.08	0.933

**Generation R (N = 569)**															
Paternal antisocial behaviour	0.07	0.04	(−0.02; 0.09)	1.43	0.153	0.06	0.04	(−0.02; 0.09)	1.35	0.178	0.08	0.04	(−0.01; 0.09)	1.42	0.158
Maternal depressive symptoms	**0.12**	**0.03**	**(0.01; 0.05)**	**2.68**	**0.008**	**0.12**	**0.03**	**(0.01; 0.05)**	**2.63**	**0.009**	**0.12**	**0.03**	**(0.01; 0.05)**	**2.68**	**0.008**
Paternal ASP * Maternal PPD											−0.03	<−0.01	(−0.02; 0.01)	−0.54	0.588

Bold values represent statistical significance *p* < 0.05.

a Adjusted for: paternal age, educational level father, infant gender (psychopathology of the other parent not included).

b As model I with additional adjustment for psychopathology of the other parent.

c As model II with inclusion of interaction term.

## 4 Discussion

In this cross country, two-cohort study, performed in the UK (Fathers Project) and The Netherlands (Generation R Study), we found a consistent positive association between paternal ASP traits and early childhood aggressive behaviour, corrected for maternal PPD-symptoms in the Generation R Study. Although this association did not reach statistical significance in the Fathers Project, the magnitude of the association was comparable to the Generation R Study. The specificity of this association was supported by the absence of an association between paternal ASP traits and early childhood internalizing behaviour problems in both cohorts. With respect to the association between maternal PPD-symptoms on early childhood aggressive behaviour the associations were less similar between the two cohorts, with a stronger association in the Fathers Project. This might partly be explained by the longer interval between the assessment of maternal PPD-symptoms and early childhood aggressive behaviour in the Generation R study, where early childhood aggressive behaviour was measured one year later than in the Fathers Project. From previous studies it is known that the effect of maternal PPD attenuates when the child grows older [23]. This attenuation effect might be less pronounced in the effect of paternal ASP traits on early childhood aggressive behaviour, since personality traits are in general more stable than depressive symptoms. Additionally, for the Fathers project recruitment was aimed at including fathers with a depression, which resulted in an oversampling of fathers with depressive disorder compared the Generation R study. It is possible that some assortative mating may occur, meaning that fathers with depression might be more likely to partner with mothers who have psychiatric co-morbidity, including antisocial personality traits [24]. This could influence the magnitude of association between maternal depressive symptoms and early childhood aggressive behavior in this study. Previous research in older children indeed showed that co-morbid maternal antisocial personality disorder increases the risk of child antisocial behavior [25].

Strikingly, in both studies there was an interaction effect between paternal ASP traits and maternal PPD-symptoms on early childhood aggressive behaviour, meaning that with higher paternal ASP traits the association between maternal PPD-symptoms and early childhood aggressive behaviour was less and vice versa. This interaction was consistent in the two cohorts and specific for early childhood aggressive behaviour, since we did not find such an interaction effect on early childhood internalizing behaviour. This negative interaction effect was not in the direction we had hypothesised, however the nature of this interaction has been described previously in a study with older children [26]. This study investigated patterns of psychopathology in families with 8-12-year-old children diagnosed with conduct problems, depression or both. The authors found an interaction effect between paternal antisocial behaviour and maternal depression on child conduct behaviour in the same direction and magnitude as we did (β= −0.18; p = 0.019). Consistent with our findings, in this study there was no interaction effect between paternal antisocial behaviour and maternal depression on child internalizing symptoms (depression scores). The authors did not discuss the possible underlying mechanisms of this finding. Here, we will make an attempt to carefully speculate. First methodological issues could be involved. Chance is not a very plausible explanation for this consistent finding in two different cohorts, but cannot be completely excluded as a possible explanation. Second, methodological flaws, e.g. reporting bias or selective attrition could underlie our observations. To rule out reporting bias, ideally reports of caregivers outside the family on early childhood aggressive behaviour should be included in the analyses. In both cohorts this information was not available and therefore we could not test the cross observer consistency. Third, the effect could be explained by other biological or environmental factors that we did not investigate, due to a lack of information in one or both studies. For example, intrauterine exposure to stress and maternal substance use, early childhood temperament, family circumstances or socioeconomic contexts could be involved.

If we consider the negative interaction between paternal ASP traits and maternal PPD-symptoms on early childhood aggressive behaviour to be a valid finding, several theoretical explanations might be possible. First, the interaction could be the result of specific patterns of family functioning and parent-child interaction in affected parents. Previous research showed that maternal PPD-symptoms, paternal ASP traits and family conflict are associated with less sensitive and more hostile interaction with infants, leading to increased risk of early childhood aggressive behaviour [27]. Our results support an independent association between paternal ASP traits and maternal PPD-symptoms on early childhood aggressive behaviour, but we did not investigate the quantity and quality of parent-child interaction and other factors that could possibly mediate the association between parental psychopathology and early childhood aggressive behaviour. It is tempting to speculate about a neutralising effect between paternal ASP traits (externalizing behaviour) and maternal PPD-symptoms (internalizing behaviour) on early childhood behaviour. For example, there is convincing evidence from previous studies that offspring from women with vulnerability for depression are at increased risk for developing depressive symptoms themselves [28]. Indeed, in our study we found an association between maternal depressive symptoms and child internalizing behaviour in both cohorts. Offspring from mothers with PPD-symptoms could benefit from genetic, parenting or environmental factors related to paternal ASP traits to make them more resilient and self-assertive in interpersonal relations in a positive sense. On the other hand, offspring of mothers without a vulnerability for depression might not benefit from paternal ASP traits behaviour as this places them at risk to become aggressive and violent themselves because they might lack a sensitivity for interpersonal relationships that is associated with depression [29]. However, these hypotheses have not been confirmed in previous research and should be tested in future studies.

### 4.1 Strengths and limitations

The main strength of this study was that we were able to compare data from two different cohorts to examine consistencies in specific associations between paternal ASP traits and maternal PPD-symptoms on early childhood aggressive behaviour. The use of cohorts from different countries (United Kingdom vs The Netherlands), different study population (higher risk sample vs population based), different measures of antisocial behaviour (self-report vs interview based) and different time of assessment of paternal antisocial behaviour (postpartum vs during pregnancy) and child age (two years vs three years), particularly strengthens the findings of similar associations. However, several limitations have to be considered. First, we were restricted in the analyses by data that was available in both cohorts. For example, in the Fathers Project no data was available on maternal ASP traits and in the Generation R Study no data was available on paternal depressive symptoms in the first months after childbirth. As a consequence we could not compare the main effects of ASP traits and PPD-symptoms between fathers and mothers on early childhood aggressive behaviour; neither could we adjust our analyses for the same pathology in the other parent. In addition, ASP traits are assessed differently in the two cohorts (the self-report Adult Selfrating (ASR) checklist in the Fathers project and the clinician administered Diagnostic Interview Schedule (DIS) in the Generation R study). Unfortunately, we are not aware of studies investigating the convergent validity of the two instruments and therefore be sure that they do not assess different aspects of antisocial behaviour. However, if we look at the effect size of paternal antisocial behaviour predicting both child aggressive behaviour and child internalizing behaviour, the magnitude of the effect is similar in the two samples. This suggests equal predictive validity. Also, in the Fathers Project, no data was available on maternal substance use (alcohol, tobacco, drugs) during pregnancy, which might have confounded the results. Second, we cannot exclude selection bias. In both samples the overall prevalence of paternal ASP traits was low, suggesting that fathers with ASP traits were less likely to participate. Also, selective attrition occurred with respect to maternal PPD-symptoms and child gender in the Fathers Project and paternal educational level in Generation R, which might have influenced the results of our study. Finally, the number of participants in the Fathers Project was relatively low with lower levels of reported ASP traits compared to the Generation R Study, which might have resulted in a lack of power to detect significant differences e.g. in the association between paternal ASP traits and early childhood aggressive behaviour.

## 5 Conclusions

The results of this study extend the current understanding of risk factors predicting early childhood aggressive behaviour. In two cohorts we found that paternal ASP traits were positively associated with early childhood aggressive behaviour, although this association did not reach significance in the Fathers Project. This association was independent from maternal PPD-symptoms and was specific for aggressive behaviour, as we did not find this association with internalizing behaviour. The magnitude of the effect of paternal ASP traits was comparable with the effect of maternal PPD-symptoms, stressing the importance of including paternal factors as well in future research on early childhood aggressive behaviour. Interestingly, we found a consistent negative interaction effect of paternal ASP traits and maternal PPD-symptoms on early childhood aggressive behaviour, meaning that with higher paternal ASP traits the association between maternal PPD-symptoms and early childhood aggressive behaviour was less. Although this negative interaction effect is perhaps unexpected and we could only speculate about the underlying mechanisms, the consistency of the findings suggests that future studies and interventions that focus on early childhood aggressive behaviour should pay careful attention to both maternal and paternal behaviour and their interactions. In addition, our study examined paternal ASP traits and maternal PPD-symptoms in a general population. We cannot infer what the results would be in a clinical population and any implications for clinical practice should be drawn with caution. However the findings do emphasize the importance of considering both mothers and fathers when considering the potential intergenerational impact of parental psychiatric difficulties. For future research it would be of particular interest to examine paternal psychopathology, including ASP traits, and its effects on early childhood development in women who present with postpartum depression in clinical practice.

## Conflict of interest

The authors declare that they have no conflict of interest.
